# The rise and fall of global health issues: an arenas model applied to the COVID-19 pandemic shock

**DOI:** 10.1186/s12992-021-00691-7

**Published:** 2021-03-29

**Authors:** Stephanie L. Smith, Jeremy Shiffman, Yusra Ribhi Shawar, Zubin Cyrus Shroff

**Affiliations:** 1 Virginia Tech 900 N. Glebe Rd., Arlington, VA 220-3-1822 USA; 2 Johns Hopkins Bloomberg School of Public Health and Paul H. Nitze School of Advanced International Studies, 615 N. Wolfe Street, Baltimore, MD 21205 USA; 3 Alliance for Health Policy and Systems Research, World Health Organization, Avenue Appia 20, 1211 Geneva, Switzerland

**Keywords:** Global health, Agenda setting, Arenas model, Coronavirus, COVID-19, HIV/AIDS, Diabetes, Alzheimer’s disease

## Abstract

**Background:**

The global health agenda is ill-defined as an analytical construct, complicating attempts by scholars and proponents to make claims about the agenda status of issues. We draw on Kingdon’s definition of the agenda and Hilgartner and Bosk’s public arenas model to conceptualize the global health agenda as those subjects or problems to which collectivities of actors operating nationally and globally are paying serious attention at any given time. We propose an arenas model for global health agenda setting and illustrate its potential utility by assessing priority indicators in five arenas, including international aid, pharmaceutical industry, scientific research, news media and civil society. We then apply the model to illustrate how the status of established (HIV/AIDS), emergent (diabetes) and rising (Alzheimer’s disease) issues might be measured, compared and change in light of a pandemic shock (COVID-19).

**Results:**

Coronavirus priority indicators rose precipitously in all five arenas in 2020, reflecting the kind of punctuation often caused by focusing events. The magnitude of change varied somewhat by arena, with the most pronounced shift in the global news media arena. Priority indicators for the other issues showed decreases of up to 21% and increases of up to 41% between 2019 and 2020, with increases suggesting that the agenda for global health issues expanded in some arenas in 2020— COVID-19 did not consistently displace priority for HIV/AIDS, diabetes or Alzheimer’s disease, though it might have for other issues.

**Conclusions:**

We advance an arenas model as a novel means of addressing conceptual and measurement challenges that often undermine the validity of claims concerning the global health agenda status of problems and contributing causal factors. Our presentation of the model and illustrative analysis lays the groundwork for more systematic investigation of trends in global health agenda setting. Further specification of the model is needed to ensure accurate representation of vital national and transnational arenas and their interactions, applicability to a range of disease-specific, health systems, governance and policy issues, and sensitivity to subtler influences on global health agenda setting than pandemic shocks.

## Background

The global health agenda is ill-defined as an analytical construct, complicating attempts by scholars and proponents to measure and make valid claims concerning the status of high-profile and neglected problems alike. Studies seeking to assess and explain differences in the agenda status of global health problems often turn to the allocation of rhetorical, policy and programmatic attention and more tangible indicators, such as human, budgetary and technical resources, to measure the agenda without clearly defining what the agenda refers to or developing methods that can be scaled up beyond the case studies that dominate this body of scholarship. Studies that assess international donor priorities by analyzing data from large aid databases form an important exception [1–3]; however, aid priorities form only a partial representation of the global health agenda. We develop and explore the potential of an arenas model to form a foundation for addressing these conceptual and measurement challenges. We leverage the emergent novel coronavirus (COVID-19) pandemic, a focusing event that provides an opportunity to investigate quick and substantial shifts (punctuation) in agendas [4–6].

We present the groundwork underpinning a collaborative effort to investigate trends in global health agenda setting under the rubric of the Global Health Agendas Project.^1^ We draw on public policy scholarship, including seminal work by Kingdon [6] and Baumgartner and Jones [4], an arenas model from sociology [7] and analyses of agenda setting in global health [8], to develop an analytical model that posits the global health agenda is formed in national and transnational arenas that overlap and interact with each other. We identify several arenas in which global health agenda setting occurs and discuss indicators that may be used to advance systematic approaches to measurement. We then apply the model to illustrate how the status of established (HIV/AIDS), emergent (diabetes) and rising (Alzheimer’s disease) issues might be measured, compared and change in light of a pandemic shock (COVID-19). We analyze the status of these global health issues in 2019 relative to their status in the year following China’s report to the World Health Organization (WHO) of a cluster of viral pneumonia cases on December 31, 2019.

Coronavirus priority indicators rose precipitously in all five arenas in 2020, reflecting the kind of punctuation often caused by focusing events or shocks [4–6] The magnitude of change varied somewhat by arena, with the most pronounced shift in the global news media arena. Priority indicators for the other conditions showed decreases of up to 21% and increases of up to 41% between 2019 and 2020, with increases in the pharmaceutical industry, media and scientific research arenas suggesting that the global health agenda expanded in some arenas in 2020—COVID-19 did not consistently displace priority for HIV/AIDS, diabetes or Alzheimer’s disease, though it might have for other issues. The study suggests that the conceptual model and measurement approach hold promise to advance knowledge useful to scholars and those working for accountability and justice on global health problems that disproportionately impact marginalized communities.

### Using an arenas model to conceptualize and measure the global health agenda

Global health includes state and non-state actors [9–12]. Kingdon [6] provides a definition of the agenda that applies to national settings: “… the list of subjects or problems to which governmental officials, and people outside of government closely associated with those officials, are paying some serious attention at any given time.” Global health scholars and proponents use indicators of serious attention in national settings, including pledges of support, policy change, budget commitments and intervention coverage by governments and other nationally engaged actors (such as donors and United Nations agencies), to assess and make claims concerning the global health agenda status of issues ranging from diarrheal diseases to undernutrition [13–18]. Assessments of the global health agenda rely in part on the extent to which problems gain traction in sovereign settings and in part on the extent to which they gain traction in arenas that transcend national boundaries.

Our analytical approach is informed by a model from sociology [4, 6, 8, 14, 19–23]. The public arenas model posits that agendas develop in collectivities of institutions not limited to governments or narrowly defined policy communities through processes of issue definition and competition for their limited resources; as such, only a limited number of issues can be on the agenda in a given arena at any time [7]. Drawing on Kingdon [6] and Hilgartner and Bosk [7], we thus conceive the global health agenda as consisting of those subjects or problems to which collectivities of actors operating nationally and globally are paying serious attention at different points in time. We assume that national and transnational arenas interact and may overlap.

Hilgartner and Bosk [7] point to social action groups, research communities, religious organizations, news and entertainment media, professional societies and branches of government as public arenas in which social problems are defined and compete for resources. Global health agenda setting arenas are comprised of collectivities of institutions that allocate and from which proponents seek attention and resources. Urgent calls to action on oral and other chronic diseases, maternal and neonatal survival, malaria, diarrheal diseases and a range of infections show that proponents often make demands in arenas comprised of political leaders, governments, multilateral institutions, the international aid community, professional groups, civil society, researchers, commercial industries (e.g. pharmaceutical and medical suppliers, food and beverage manufacturers), and groups in interdependent sectors (e.g. water and sanitation, education, trade), among others [24–29]. Mass media is also recognized as an important arena for global health agenda setting, with headlines shaping public awareness and policymaker decisions [30].

In their calls to action, proponents often implore actors in various agenda setting arenas to invest resources in: recognizing and engaging global health problems; providing leadership and coordination; advancing disease surveillance, prevention and control; developing and executing plans, policies and programs; allocating financial, technical and human resources; setting goals and supporting robust systems for monitoring and accountability; building health system capacity; conducting research that is relevant to low- and middle-income countries; making medicines and technologies widely accessible; and protecting patients and consumers [24–29]. Global health agenda setting scholars often turn to a patchwork of measurement approaches that use more and less reliable and comparable indicators such as these to assess the agenda status of health problems nationally and globally [13, 14, 18, 31–33]. A public arenas model offers advantage in part by narrowing the unit of analysis to arenas in which global health agenda setting occurs and for which reliable and comparable indicators are available if underutilized.

Advances that have been made in measuring priorities in the international aid arena by analyzing financial data from the WHO Global Health Expenditure Database, Institute for Health Metrics and Evaluation [34] and the OECD’s Creditor Reporting System Database [2] suggest that taking arenas as the central unit of analysis offers advantages. Actors comprising different arenas offer and are asked to prioritize problems by allocating fairly distinctive forms of attention and resources, pointing to indicators that may be used to measure the status of issues in various arenas. For instance, actors comprising the international aid arena offer and are asked to provide development assistance for health, the news media column space and industry arenas research, development and product supply.

Several arenas and indicators that are closely associated with them may be analyzed separately and collectively to gain a more nuanced understanding of which problems are on the global health agenda at any given time, and to identify leading, lagging and irrelevant arenas—which may vary by the type of global health issue. For instance, emergent infectious disease outbreaks may commonly attract headlines in the news media arena. Meanwhile, the status of nonclinical issues like road traffic injuries and health system management may persistently lag in the pharmaceutical industry arena, reflecting limited need and relevance.

We use a set of five arenas that extend beyond national boundaries (i.e., transnational arenas), including international aid, news media, pharmaceutical industry, scientific research and civil society to illustrate the utility of an arenas model for global health agenda setting and a measurement approach that employs reliable and comparable indicators. We focus on transnational arenas for global health agenda setting because approaches to conceptualizing and measuring the status of issues in them is underdeveloped compared to national arenas. Scholarship informing the set of preliminary arenas and potential indicators is discussed in the paragraphs that follow, with those indicators incorporated into our exploratory study highlighted in Table 1.

**Table 1:** Arenas, indicators and data sources used in this study

Arenas	Indicators	Data sources
International aid	Development Assistance for Health	Financing Global Health Visualization tool (Institute for Health Metrics and Evaluation, 2019)
International aid	Clinical trials (industry-sponsored)	The Australian New Zealand Clinical Trials Registry (anzctr.org.au) and ClinicalTrials.gov (sponsored by the U.S. government)
International aid	Bibliographic trends	PubMed database for overall trends; *The New England Journal of Medicine* (Advanced Search tool at https://www.nejm.org/ medical-search); *The Lancet* (Science Direct)
	Clinical trials (not industry-sponsored)	The Australian New Zealand Clinical Trials Registry (anzctr.org.au) and ClinicalTrials.gov (sponsored by the U.S. government)
News media	Publishing trends	Access World News database; Factiva news database
Civil society	Community mobilization	Fundraising or programmatic activity among nonstate organizations in offi cial relations with WHO in 2020

#### International aid arena

Development assistance for health (DAH), which has grown substantially since 1990 and reached $41 billion in 2019 [34], is a common status indicator in the international aid arena [1–3]. Analyses show that HIV/AIDS, which accounted for nearly a quarter of DAH in 2019, surged ahead in the competition for financial resource allocations in the international aid arena over the past two decades [35], displacing aid to malaria and the broader health sector in the mid-2000s [36]. DAH has also been used as an indicator of donor priority for maternal and child survival relative to newborn survival [37, 38].

#### Industry arenas

The status of global health problems in commercial industrial arenas is often crucial, shaping access to preventive and therapeutic drugs, medical equipment and technologies, and environmental risks. For instance, though the status of HIV/AIDS has since risen, doubts that a market for antiretroviral drugs existed in low-income countries suppressed investment in the pharmaceutical industry arena in the 1990s and early 2000s [39]. In addition, research shows that diseases prevalent in low-income countries compete with those prevalent in high-income countries for clinical drug trial investments in the pharmaceutical industry arena, with cancer treatments leading the pack between 2006 and 2011 [40]. Trends in industry-funded registered clinical drug trials may thereby form an important indicator of status in the pharmaceutical industry arena. The relevance of other industry arenas and indicators should also be considered. For instance, the hygiene product industry arena plays into the status of menstrual hygiene management as a global public health issue [41]. So do the transnational tobacco and alcohol industry arenas for non-communicable diseases [42].

#### Scientific research arena

Global health problems are defined and compete for resources among collectivities of scientific researchers and institutions that generate evidence of their nature, severity and tractability, with implications for other arenas. For instance, growing evidence of the toll and tractability of maternal and neonatal mortality resulting from prioritization among researchers helped these issues gain traction in national and aid arenas [38]. Bibliographic trends in medical and public health journals, which have been used to track priority for diarrheal diseases and maternal health interventions among researchers and funders, comprise an important indicator of priority in the scientific research arena [13, 43]. Data on dissertations, sponsored research and non-industry funded clinical drug trials might be used as indicators of emergent and current research attention that is not well captured by recent bibliographic trends due to the time it takes to conduct and publish research [13, 40].

#### News media arena

News media, including television news, magazines, newspapers and radio, are among Hilgartner and Bosk’s [7] original arenas, with column inches and minutes of air time serving as index measures. Publishing trends indicate that news media privilege certain health issues and portrayals, informing public and elite policy decisions on such issues as diabetes [44], childhood obesity [45], and reproductive health [46]. Publishing trends have also been used to show that the status of Global Fund diseases, including HIV/AIDS, tuberculosis and malaria, is much higher than lower funded and higher burden childhood pneumonia, diarrhea and measles in the news media arena [47].

#### Civil society arena

Referring to “the broad spectrum of voluntary associations that are entirely or largely independent of government and that are not primarily motivated by commercial concerns” [48], civil society has grown in prominence and centrality over the past few decades due to its expanding role in generating international support for such critical public health issues as HIV/AIDS, reproductive health and tobacco control [49–51]. Surveys of members of The World Federation for Mental Health and European Public Health Alliance have been used to assess priorities in the segments of the civil society arenas concerned with these issues [52, 53]. And widespread community mobilization has been cited as an indicator of priority for children affected by HIV/AIDS [54]. These measurement approaches are not viable for this study, which seeks to capture shifts in the global health agenda during a quickly evolving emergent disease pandemic. We settled on an imperfect solution, observing indicators of civil society mobilization on our issues via a purposeful sample of nonstate organizations’ websites.

To summarize, we propose that global health agenda setting occurs in several interacting national and transnational arenas. A contribution of an arenas model for global health agenda setting is the potential use of a measurement approach that involves the systematic observation and analysis of several reliable and comparable, if preliminary, indicators of priority within distinctive arenas. The model and measurement approach have strong potential to enhance support for inferences about a fragmented global health agenda. The measurement approach is likely to benefit from complementary case study research, which may be better suited to uncovering indicators that are difficult to assess using quantitative methods (e.g., emergent disease threats being discussed among isolated clusters of specialists), the nuances of programmatic attention to established versus emergent health conditions, and causal mechanisms that underly punctuations and stability [4, 19, 55]. Steps should also be taken to ensure that the overall measurement strategy avoids crowding out other forms of knowledge that are not readily quantifiable [56].

## Methodology

Our exploratory study employs a pre-post design, asking: What was the relative agenda status of a subset of set of global health issues in five transnational arenas in 2019? How did priority for these issues change between 2019 and 2020, as the COVID-19 pandemic emerged? We use the constrained time period to explore the propositions that: (1) agendas in some arenas are likely to exhibit punctuations that are more proximate to stimuli and more pronounced than those in other arenas and (2) shocks are likely to displace priority for other issues. We expect to find quicker and stronger responsiveness to the pandemic shock and displacement effects on less established issues in the news media arena (which is characterized by 24-h news cycles) compared to the scientific research arena (which typically involves relatively long periods of time to design, fund, conduct and publish studies), for instance.

We explore the agenda status of four issues selected for varying salience to the global health agenda setting arenas included in the study—one long established on the global health agenda (HIV/AIDS), one emergent (diabetes, which has risen as a global health priority over the past two decades [23]), one newly rising (Alzheimer’s disease, with increasing calls for prioritization in recent years [57, 58]) and one a significant shock (COVID-19). Inclusion of COVID-19 and other coronaviruses (comparative predecessors) promises insights to the impacts of a pandemic shock versus status quo conditions in the arenas. Issues selected for inclusion feature significant mortality burdens (Table 2) and ongoing needs for development and distribution of preventive and therapeutic treatments. We also considered the distinct naming conventions of these diseases, which facilitates data collection using a range of databases. Coronavirus and COVID-19 data are collected and analyzed separately to avoid problems caused by different and overlapping terms of reference used in the context of an evolving pandemic—COVID-19 was only officially named by WHO on 11 February 2020.^2^

**Table 2:** Global health agenda salience, issues and mortality burdens

Agenda salience	Diseases	Number of Deaths
Established	HIV/AIDS	954,500
Emergent	Diabetes	1,370,000
Rising	Alzheimer’s disease and other dementias	2,515,000
Shock	COVID-19° (2020)	~ 1,800,000

Sources and Notes: (1) °COVID-19 deaths are for 2020 [59]. (2) Estimates for all other diseases are at 95% Uncertainty Interval [60].

Data collection and analysis procedures used in this study are summarized below for each arena. Data sources were selected for availability of reliable data for 2019 and 2020, transparency (with most accessible without a paywall), and in the cases of DAH, clinical trials and PubMed following precedents [2, 3, 13, 36, 40, 43]. Due to differences between arenas and corresponding indicators, methods for each vary.

### International aid arena

We collect and analyze DAH from all sources (US$41 billion) in 2019 as tracked by the Institute for Health Metrics and Evaluation [34]. We also analyze data from the same database for the four largest independent sources of aid in 2019, including the United States (US$12 billion), United Kingdom (US$3.5 billion) and Germany (US$2.1 billion) and the Bill & Melinda Gates Foundation (US$3.9 billion), for our 2020 comparison since data on DAH from all sources was not available for the time period. We use the Financing Global Health Visualization tool [34] to compare allocations to HIV/AIDS, “Other infectious diseases-other” (which is a subcategory of “Other infectious diseases” that includes 16 named and other unnamed diseases, including coronaviruses and excluding malaria, Ebola, Zika, antimicrobial resistance, health systems support and human resources) and “Other NCDs” (which includes diabetes and Alzheimer’s disease and excludes mental health, health systems support, human resources and tobacco) [61]. We maintain category names from the database, particularly “Other infectious diseases-other,” for specificity and to facilitate cross-reference with the database.

COVID-19 assistance pledged by the U.S., U.K., Germany and Gates Foundation during the first quarter of 2020 was obtained from press and news releases published on the respective organizations’ websites. These pledges are compared to the quarterly average of their DAH allocations to “Other infectious diseases-other” in 2019 and the percent difference between time periods.

### Pharmaceutical industry

Registered clinical trials data for 2019 and 2020 were obtained from two databases that allow for systematic selection of studies funded by industry and that feed into the WHO’s International Clinical Trials Registry Platform database in 2019 and 2020. The Australian New Zealand Clinical Trials Registry (anzctr.org.au) and the widely used ClinicalTrials.gov (sponsored by the U.S. government) were searched using the following terms: coronavirus; COVID-19; diabetes; Alzheimer*; and HIV (capturing HIV and AIDS). Total studies funded by industry and the percent change between time periods are compared.

### Scientific research

Data on bibliographic entries were obtained using searches of four databases. A PubMed database search provides an overview of bibliographic trends in medical and public health fields [13]; absolute and percent change in bibliographic entries by issue in 2019 and 2020 are compared. Searches in two medical and two global public health journals with broad scope and that according to Web of Science InCites Journal Citation Reports for 2018 are widely cited, show emergent trends by issue among leaders in the first quarter of 2020, which are compared to the quarterly average in 2019. *The New England Journal of Medicine* (Impact factor 70.670) was searched using the Advanced Search tool on the journal’s website. Searches of *The Lancet* (Impact factor 59.102) and *The Lancet Global Health* (Impact factor: 15.873) used the Science Direct database. A topic search in the Web of Science Core Collection database was used for the *Bulletin of the World Health Organization* (Impact factor 6.818). Different databases were searched to obtain the most accurate and up-to-date results for the journals. Because this study’s purpose is to capture the agenda status of issues in the scientific research arena broadly, these journals were selected over those oriented to disease-specific specialist communities.

Data on registered clinical trials were obtained from two databases that allow for systematic search and selection of studies by health condition and time period and feed into the WHO International Clinical Trials Registry Platform to provide a sense of agenda status during the pre-publication stage—as an indicator of emergent agenda status. These included: Australian New Zealand Clinical Trials Registry and ClinicalTrials.gov (sponsored by the U.S. government), with differences between 2019 and 2020 compared.

### News media

Publishing data were obtained via an Access World News database search covering the following regions and corresponding number of sources: Africa (644); Asia (915); Australia/Oceania (750); Europe (1229); Middle East (428); North America (9723); South America [50]. The Factiva news database was searched to identify publishing trends in *The Guardian* (U.K.) and *The New York Times*, two global news media leaders.

### Civil society

We selected a subset of transnational civil society that is formally engaged in the international system for this preliminary inquiry. We surveyed the websites of the 132 civil society organizations (CSOs) in official relations with WHO (2020); these organizations represent a prominent and high-capacity segment of civil society, limiting the generalizability of results. We surveyed and coded for evidence of fundraising or program activity on HIV/AIDS, other infectious diseases, diabetes, Alzheimer’s disease, and the novel coronavirus (COVID-19) as indicators of priority during the first week of April and October of 2020. Announcements (e.g., meeting cancellations) and links to external media and the WHO website alone were not counted.

## Results

The novel coronavirus (COVID-19) punctuated the agenda in every arena during 2020. As expected, we find some variation in the timing and magnitude of shifts across arenas. The status of the pandemic rose quickly among leading news media and medical journals, with COVID-19 also rising precipitously in the five broader arenas during 2020. We present detailed findings concerning the relative status of the set of established, emergent and rising global health problems and coronaviruses (including the COVID-19 shock) in 2019 and 2020 by arena in this section, with further comparison of arenas in the discussion section. We report results for “coronavirus” and “COVID-19” separately in some arenas to reflect different problem frames, to compensate for lag time between problem recognition and naming COVID-19 in mid-February, and to avoid over-representation that combining results may produce.

### International aid arena

**Fig. 1:**
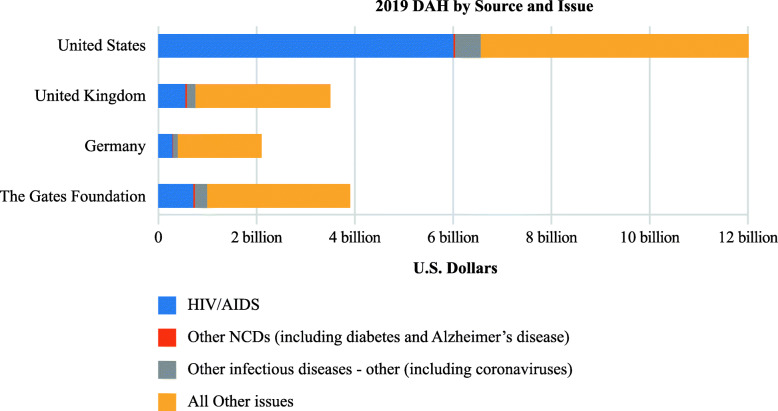
2019 DAH by Source and Issue

In 2019, HIV/AIDS received a quarter of all DAH (US$9.5 billion of US$41 billion) [34]. “Other infectious diseases-other” (including coronaviruses) trailed HIV/AIDS by a wide margin, receiving US$1.7 billion, while Other NCDs (including diabetes and Alzheimer’s disease) received US$730 million. Figure 1 shows the same ordering of priorities among each of the four largest independent sources of aid in 2019.

**Fig. 2:**
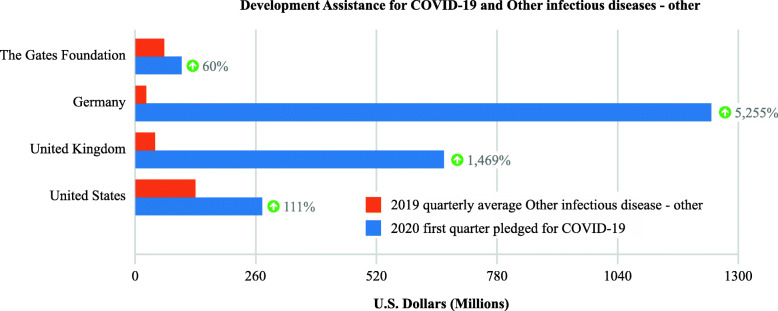
Absolute and percent difference between DAH for COVID-19 and “Other Infectious Diseases–Other”

Pledges of DAH to COVID-19 in early 2020 indicate the issue’s status rose quickly among the four largest independent donors. Pledges to COVID-19 in the first three months of 2020 outpaced DAH to “Other infectious diseases-other” and Other NCDs during all of 2019, with only HIV/AIDS attracting more. Pledges to COVID-19 in the first quarter of 2020 were nearly 16 times higher in the U.K., more than 23 times higher in Germany, two times higher in the U.S. and 1.6 times higher for The Gates Foundation than their quarterly DAH was for “Other infectious diseases-other” in 2019 (Fig. 2). Reinforcing these findings, the Organisation for Economic Cooperation and Development (OECD) published a report indicating that 28 Development Assistance Committee members allocated an estimated US$12 billion (including US$7 billion new or extra-budgetary) for COVID-19 response in developing countries in 2020 [62].

### Pharmaceutical industry arena

**Fig. 3:**
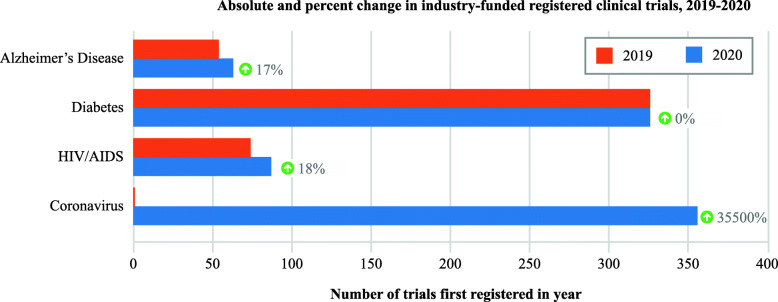
Absolute and percent change in registered clinical trials

Indicative of the order of priorities in the pharmaceutical industry arena in 2019, industry funded more than four times as many clinical trials addressing diabetes (326) as HIV/AIDS (74) and six times more than those addressing Alzheimer’s disease (54). Industry funding for trials addressing coronavirus (1, a study COVID-19 was added to in 2020) lagged far behind. Priorities shifted in 2020, with trials pertaining to COVID-19 (826) and coronavirus (356) rising to the top, followed by diabetes (326), HIV/AIDS (87) and Alzheimer’s disease (63). Figure 3 shows the number of studies supported by industry funding by issue and the percent change between time periods.

### Scientific research arena

Using clinical trials without industry funding as an indicator of emergent scientific research attention shows diabetes (1034) leading HIV/AIDS (382) by a wide margin, followed by Alzheimer’s disease (178) and coronavirus (5, all addressing Middle East Respiratory Syndrome). COVID-19 (3488) and coronavirus (1039; 20,680% increase) rose to the top in 2020, followed by diabetes (990; 4% decrease), HIV/AIDS (315; 18% decrease) and Alzheimer’s disease (176; 1% decrease).

**Fig. 4:**
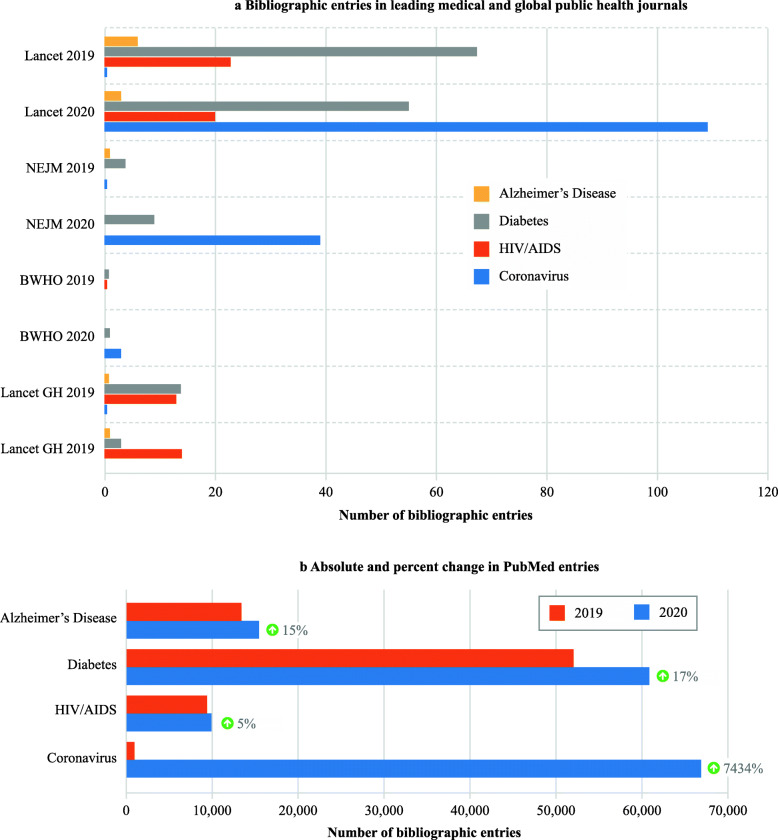
**a** Bibliographic Entries in High-Impact Medical and Global Public Health Journals. Note: **a** compares the quarterly average of publications in 2019 to total publications in the first quarter of 2020. **b** Absolute and percent change in PubMed entries

Turning to leading medical journals, which may respond more quickly than the arena writ large, diabetes led bibliographic entries in *The Lancet* and *New England Journal of Medicine* in 2019 while coronavirus and COVID-19 led in early 2020 (Fig. 4a). Diabetes also led in *The Lancet Global Health* and *Bulletin of the World Health Organization* in 2019. HIV/AIDS became the lead issue in *The Lancet Global Health* in the first quarter of 2020, with coronavirus and COVID-19 yet to appear in an issue, while coronavirus took the lead in the *Bulletin* during the same period.

Taking bibliographic trends in PubMed as a broader indicator of agenda status in the scientific research arena, diabetes led in entries by a wide margin in 2019—fourfold over Alzheimer’s disease and fivefold over HIV/AIDS (Fig. 4b). Bibliographic entries in PubMed increased for all four issues in 2020, with the pandemic shock reflected in an increase of more than 7400% for coronaviruses to nearly 69,000 entries (COVID-19 had 90,113 entries)—surpassing diabetes’ by a more than 10% margin. Coronavirus and COVID-19 entries had lagged in the first quarter of 2020, with nearly 2500 entries for each compared to approximately 15,000 entries for diabetes.

### News media arena

**Fig. 5:**
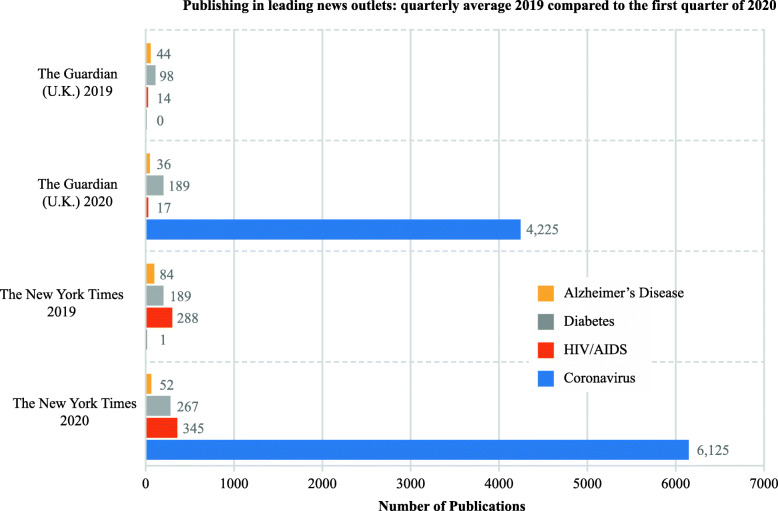
Publishing in Leading News Outlets

Access World News Database search results show that diabetes (275,732 publications) and Alzheimer’s disease (176,775) enjoyed higher status than HIV/AIDS (32,176) and coronaviruses (1204) in 2019. Publishing on coronavirus (9,107,231) and COVID-19 (10,781,286) exploded in 2020, dwarfing coverage of other issues. Nonetheless, publishing on diabetes increased by 26% and HIV/AIDS by 41% in 2020. Coverage of Alzheimer’s disease decreased by 21%. Leading outlets reflect broader trends, with *The New York Times* and *The Guardian* shifting coverage toward coronavirus and COVID-19 in early 2020—both published approximately 20 times the number of articles on coronavirus compared to the next issue (diabetes) during this period (Fig. 5).

### Civil society arena

**Fig. 6:**
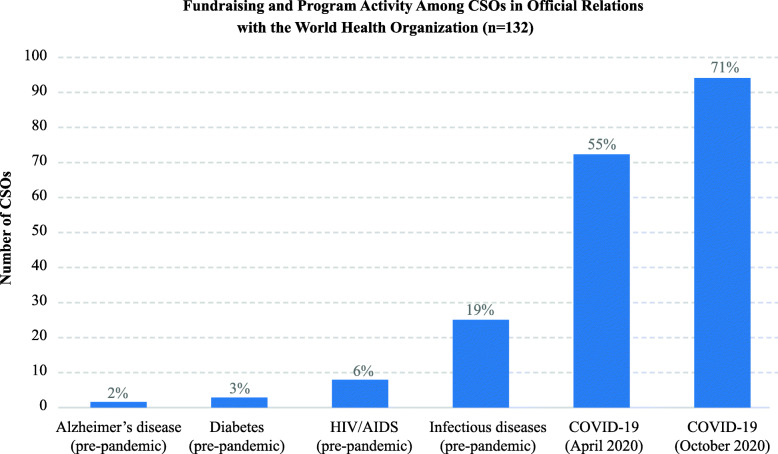
Fundraising and Program Activity Among CSOs in Official Relations with the World Health Organization (*n* = 132)

As indicated by fundraising and program activity, infectious diseases as a broad category were on the agendas of just under 20% of the 132 CSOs in official relations with the WHO pre-pandemic. HIV/AIDS (6%), diabetes (3%) and Alzheimer’s disease (2%) were represented in relatively small numbers. More than half rolled out fundraising campaigns and program activities, including technical guidance for their constituencies, on COVID-19 in the first quarter of 2020 (Fig. 6). More than 70% had done so by the beginning of the fourth quarter, indicating the issue rose quickly and steadily in this arena during 2020.

## Discussion

Despite strong and plentiful calls to put a wide range of significant problems that transcend geographic and political boundaries on the global health agenda—to reorder the global health agenda—the concept and its measurement have remained ill-defined. An arenas model for global health agenda setting points to solutions to conceptual and measurement challenges that often undermine the validity of claims concerning the status of problems and contributing causal factors. We illustrate the utility of the conceptual model by systematically measuring a set of indicators associated with five vital transnational arenas for global health agenda setting, revealing significant shifts between 2019 and 2020. The novel coronavirus punctuated the agenda in all arenas in 2020 (Table 3).

**Table 3:**
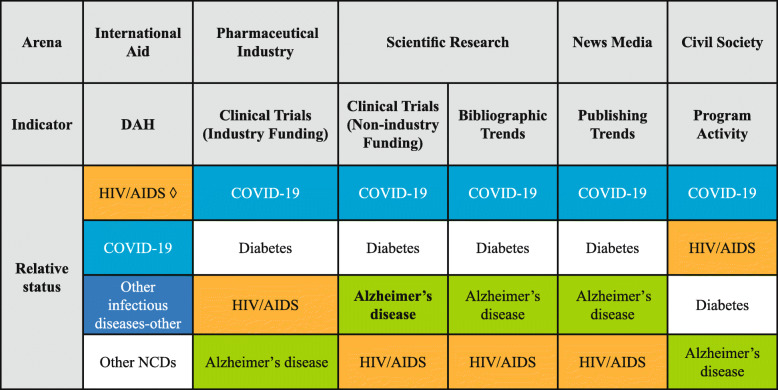
Relative status by arena, indicator and issue in 2020

Notes: Bold = moved up from 2019 status; ◊ = no change. Status in the international aid arena compares COVID-19 pledges from the U.S., U.K., Germany and the Gates Foundation to their 2019 aid allocations to other issues.

The magnitude of punctuation varies somewhat by indicator, reflecting differences in agenda setting dynamics within each arena. The largest punctuation occurred in the news media arena with coverage of COVID-19 outpacing the next issue (diabetes, which led in 2019) 26 to 1 in 2020. Importantly, coverage of diabetes and HIV/AIDS (emergent and established issues) also increased in 2020 while coverage of Alzheimer’s disease (a more recently rising issue) declined. This suggests the possibility that the COVID-19 focusing event had crowding in (e.g., increasing priority for emergent and established issues) and crowding out (e.g., displacing a recently rising issue) effects in the news media arena. It may also have had broader additive effects, growing the overall agenda for global health issues.

The agenda also shifted significantly in the international aid arena. Pledges to COVID-19 in early 2020 from the four largest independent sources of aid outpaced DAH to “Other infectious diseases-other” in all of 2019 by a two to one ratio and trailed only DAH to HIV/AIDS during the same time period. The OECD’s report of US$7 billion new or extra-budgetary allocations to COVID-19 relief from DAC members in 2020 is suggestive of additive effects on the agenda for global health in the international aid arena; the US$5 billion that was re-allocated toward COVID-19 suggests priority for some other issues was displaced by the pandemic.

In the pharmaceutical industry arena, priority for coronaviruses spiked as more clinical trials were registered for COVID-19 than for diabetes (the emergent 2019 leader) in 2020. The number of industry-funded clinical trials addressing diabetes held steady in 2020 while they increased for HIV/AIDS and Alzheimer’s disease. COVID-19 appears not to have displaced priority for this set of issues, though it might have done so for issues and time periods that are beyond the scope of this study.

COVID-19 also led non-industry funded trials (diabetes was the 2019 leader) and became the lead issue at top medical journals early in 2020, indicating the emergent problem rose to prominence quickly in the scientific research arena. Non-industry funded trials and entries at top medical journals were down for the other three issues compared to their 2019 levels, possibly capturing proximate priority displacement effects. COVID-19 also came to dominate bibliographic entries in the PubMed database; that entries were also up for the other three issues is likely an artifact of their pre-pandemic agenda status in the scientific research arena.

Lastly, a subset of civil society organizations, which we explore in a preliminary way as a proxy for the civil society arena, incorporated COVID-19 into their fundraising and program activities at a rate more than three times that of the broader category of infectious diseases pre-pandemic. COVID-19 rose on the agenda in the civil society arena; possible effects on priority for the other issues remain unclear due to limitations of the data. Collectively, our findings point to major shifts in the global health agenda between 2019 and 2020.

The quick and strong magnitude of shifts observed in the set of global health agenda setting arenas included in this study mirror the kinds of punctuations often caused by focusing events that interrupt long periods of relative stability in the policy process [4–6]. The novel coronavirus pandemic offers a rare opportunity to observe such shifts in the global health agenda—less widespread and less deadly infectious and noncommunicable disease outbreaks are unlikely to trigger the same magnitude of change in such a short period of time, though there may be some variation by arena and some of the changes we observed are likely due to other stimuli (e.g., new scientific discoveries). It is therefore important to investigate the utility of the model under differing conditions and over longer periods of time. It is also imperative to develop indicators that better represent certain arenas, including broader swaths of the civil society and international aid arenas, for instance. Nonetheless, grounded in an arenas model, our measurement approach captures the effects of agenda setting dynamics in diverse arenas, reflecting the swiftness of news cycles and laborious nature of the research publishing endeavor. It also reflects temporal change, suggesting an arenas model for global health may have utility for studies seeking to assess and explain the differential status of a broader range of issues over extended periods of time.

## Conclusions

Why do some issues rise on the global health agenda while others remain neglected? What does it mean for an issue to be prioritized or neglected? These are crucial questions for proponents and scholars alike. The agenda must be defined and measured to provide robust answers to these. This study represents a foundational step toward such specification.

We contribute an analytical model that posits the global health agenda is formed in transnational and national arenas that overlap and interact with each other. We apply the model to five crucial arenas for global health agenda setting—international aid, pharmaceutical industry, scientific research, news media and civil society—illustrating how the status of established (HIV/AIDS), emergent (diabetes) and rising (Alzheimer’s disease) issues might be measured, compared and change in the context of a pandemic shock (COVID-19). As expected, coronavirus indicators spiked in all arenas. Priority decreased for some issues (publishing on recently rising Alzheimer’s disease decreased by 21% in the news media arena), suggesting possible displacement effects. However, priority was largely stable or on the rise for most issues across arenas in 2020, including publishing on diabetes and HIV/AIDS in the news media arena and clinical trials addressing Alzheimer’s disease in the pharmaceutical industry arena—these increased by more than 25%. The prevalence of increases in priority indicators suggests that multiple arenas expanded their agendas for global health in the wake of COVID-19. The arenas model and illustration presented here provide a glimpse into ways in which the status of established, emergent and rising global health issues may vary by arena and in the wake of a pandemic shock.

The arenas model for global health agenda setting and measurement approach presented in this article raise several vital questions for future research, including:

- How can transnational and national arenas be further integrated into the model to better reflect the global health agenda? How can indicators be developed to better represent vital arenas, particularly global civil society and lower-income countries?
- Which other transnational arenas are integral to shaping the status of global health problems? Do some arenas matter more for some kinds of issues than others? Are some arenas leaders and others followers? Does it depend on the type of problem? How can interaction effects between transnational and national arenas be conceptualized and measured?
- How do the model and measurement approaches need to be adapted for issues that are not easily defined by a disease category, such as environmental and sexual and reproductive health? What about health systems, governance, and equity issues?
- How might tools such as machine learning and natural language processing be used to improve measurement and extend it over long periods of time?
- Is the measurement approach sensitive enough to detect subtler influences on global health agenda setting? Does it reflect the impacts of smaller scale infectious disease outbreaks? Of other diseases and conditions? Does it reflect the effects of political transitions, emergent evidence of tractability, champions, or civil society movements?
- What magnitude of change is normal and what reflective of major punctuations? Is the agenda more elastic in some arenas than others? If so, what are the implications for agenda setting in other arenas?
- Under what conditions does the global health agenda expand and contract? Under what conditions do global health issues and agendas displace other issues and agendas? How do these dynamics differ by arena?

More work is needed to inform and later to validate the conceptual model and measurement approach proposed here. Geared to advancing knowledge of global health agenda setting dynamics and trends, such work would be useful to scholars assessing the status of issues and to groups demanding social justice and accountability on health problems that disproportionately impact marginalized groups worldwide.

## Data Availability

The publicly accessible data that support the findings of this study are available from PubMed (pubmed.gov), the Institute for Health Metrics and Evaluation (https://vizhub.healthdata.org/fgh/), the Australian New Zealand Clinical Trials Registry (anzctr.org.au), ClinicalTrials.gov, *The New England Journal of Medicine* (https://www.nejm.org/medical-search) and the websites of the non-state organizations in official relations with WHO during 2020 (available from the first author upon request). Data supporting the findings of this study with respect to *The Lancet*, *The Lancet Global Health* and *Bulletin of the World Health Organization* (ScienceDirect) and media publishing trends (Access World News and Factiva news databases) were used under license for the current study, and so are not publicly available.

## Footnotes

1 The Global Health Agendas Project is informed by but not affiliated with the Policy Agendas Project (Baumgartner and Jones, 1993) and Comparative Agendas Project (https://www.comparativeagendas.net/).

2 https://www.who.int/emergencies/diseases/novel-coronavirus-2019/technical-guidance/naming-the-coronavirus-disease-(covid-2019)-and-the-virus-that-causes-it
